# Making gene drive biodegradable

**DOI:** 10.1098/rstb.2019.0804

**Published:** 2020-12-28

**Authors:** Josef Zapletal, Neda Najmitabrizi, Madhav Erraguntla, Mark A. Lawley, Kevin M. Myles, Zach N. Adelman

**Affiliations:** 1 Department of Industrial and Systems Engineering, Texas A&M University, College Station, TX 77843, USA; 2 Department of Entomology and Agrilife Research, Texas A&M University, College Station, TX 77843, USA

**Keywords:** gene drive, mitigation strategy, self-elimination mechanism, mosquito, risk assessment, clustered regulatory interspaced palindromic repeat

## Abstract

Gene drive systems have long been sought to modify mosquito populations and thus combat malaria and dengue. Powerful gene drive systems have been developed in laboratory experiments, but may never be used in practice unless they can be shown to be acceptable through rigorous field-based testing. Such testing is complicated by the anticipated difficulty in removing gene drive transgenes from nature. Here, we consider the inclusion of self-elimination mechanisms into the design of homing-based gene drive transgenes. This approach not only caused the excision of the gene drive transgene, but also generates a transgene-free allele resistant to further action by the gene drive. Strikingly, our models suggest that this mechanism, acting at a modest rate (10%) as part of a single-component system, would be sufficient to cause the rapid reversion of even the most robust homing-based gene drive transgenes, without the need for further remediation. Modelling also suggests that unlike gene drive transgenes themselves, self-eliminating transgene approaches are expected to tolerate substantial rates of failure. Thus, self-elimination technology may permit rigorous field-based testing of gene drives by establishing strict time limits on the existence of gene drive transgenes in nature, rendering them essentially biodegradable.

This article is part of the theme issue ‘Novel control strategies for mosquito-borne diseases'.

## 1. Introduction

Gene drive systems have long been sought by the vector biology community in order to drive pathogen-resistance traits into mosquito populations to combat malaria and dengue [1]. Following successful demonstrations that some homing endonuclease genes (HEGs) can edit the genomes of *Anopheles* mosquitoes [2,3], an HEG (I-SceI) was found to be capable of driving into a cage population of *Anopheles gambiae* when its target site was engineered in the mosquito genome [4], confirming earlier predictions [5]. The rapid development of reagents for clustered regulatory interspaced palindromic repeat (CRISPR) gene editing [6–8] introduced a new programmable nuclease that was far simpler to target at new sites than HEGs. CRISPR was thus rapidly seen as an alternative programmable nuclease that could replace HEGs in gene drive [9], with successful CRISPR-based gene drive described in *Drosophila* [10] and Anopheline mosquitoes [11,12] not long after. The relative simplicity of the CRISPR/Cas9 homing-based gene drive approach elevated concerns about gene drive technology, triggering the rapid development of a report by the National Academy of Science [13]. While genetic resistance to gene drive approaches was observed in early proof-of-principle experiments [14–16], it is now clear that selection for genetic resistance can be avoided by using conserved target sequences. Indeed, a recent gene drive approach targeting a highly conserved region of the *doublesex* (*dsx*) gene critical for female mosquito development successfully eliminated caged populations of *A. gambiae* in fewer than 10 generations, with selection unable to act on resistance alleles [17].

The development of self-perpetuating CRISPR-based gene drive approaches has led to calls for increased regulatory capacity [18], institutional oversight [19,20] and responsible governance [21,22], as such gene drive transgenes could become established in the wild through the accidental release of just a few individuals during testing [23,24]. As a result, gene drive technology has arrived at a seemingly impossible paradox: how to safely field-test a system that by its very nature may permanently alter a natural population. Strategies to split a gene drive transgene into multiple pieces are predicted to limit the spatial distribution of the invading gene [25,26], but do not prevent the long-term establishment of one or more transgenes in nature, even during field-testing when potential hazards are unknown. Remediation in the form of additional large-scale mosquito releases are a potential reversal mechanism for current gene drive approaches [25,27]. However, this is far from ideal, as a field trial to evaluate a gene drive transgene may be forced to conclude abruptly owing to factors outside the control of the research team (natural disaster, armed conflict, political change, etc.).

The unwanted persistence of transgenic material is not a unique problem to gene drive or vector biology, and efforts to ensure the removal of unwanted transgenes have been ongoing in both agricultural [28,29] and human gene therapy [30–32] applications. We reasoned that approaches developed in these disciplines but applied in the context of gene drive might make it possible to pre-programme the reversion of a gene drive transgene to a non-transgenic allele through selective excision of all transgene material in a manner than can be repressed during a trial. To evaluate whether such technologies are worth pursuing in the context of homing-based gene drive, we developed a series of deterministic models to calculate the dynamics of the spread and persistence of a gene drive transgene in a population in the presence of what we refer to as a ‘self-elimination mechanism’ (SEM). Self-eliminating approaches were found to provide temporal control, rapidly reversing the invasion of a gene drive transgene even at very low rates of effectiveness (less than 10%) while tolerating substantial rates of failure. Stacking multiple self-elimination approaches together provided an additional layer of spatial control and could potentially serve as a form of biocontainment, preventing the invasion of gene drive transgenes into native populations during the evaluation phase. Based on these results, we suggest that homing-based gene drive transgenes can be engineered to be completely biodegradable in the environment, obviating any need for bioremediation and allowing extensive risk assessment prior to the consideration for widespread use.

## 2. Results

**Figure 1.**
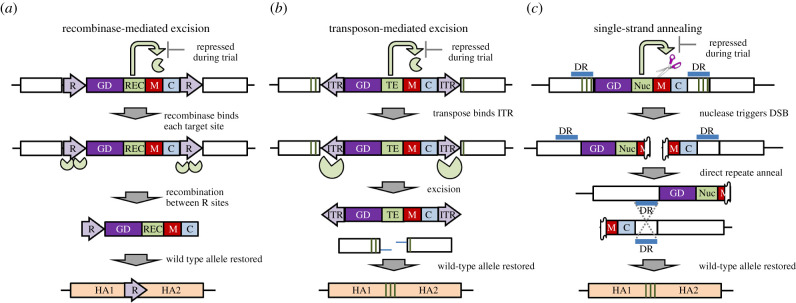
Potential mechanisms for a self-eliminating CRISPR/Cas9-based gene drive (GD). The GD transgene is linked to marker (M) and cargo (C) genes, with the SEM based on: (*a*) a site-specific recombinase (REC) and corresponding recombination (R) sites, (*b*) an integration-defective transposase (TE) and corresponding inverted terminal repeats (ITRs) or (*c*) SSA-based DNA repair initiated by a nuclease (NUC) and enabled by direct repeats (DR). In all cases, the disrupted, non-functional host gene is indicated by white boxes, with the restored, functional gene indicated by filled (peach) boxes. Vertical green bars indicate the recoded sequences rendering the restored gene resistant to the GD.

We identified at least three independent mechanisms that could be incorporated into a homing-based gene drive to limit its persistence in nature (figure 1). In case 1, the gene drive transgene, any associated marker(s), and cargo genes would be accompanied by a gene encoding a recombinase, and the entire cassette flanked with corresponding recombination sites. Expression of the recombinase would result in intramolecular recombination between the two flanking regions resulting in the excision of the intervening gene drive transgene, as well as all other transgenes, and restoration of the host allele (figure 1*a*). In case 2, the gene drive transgene and associated marker/cargo genes are accompanied by a gene cassette encoding an integration-deficient transposase [30], and flanked with corresponding inverted terminal repeats (ITRs, figure 1*b*). Expression of the transposase results in its binding to the ITRs and initiation of targeted double-stranded DNA breaks, resulting in the loss of all transgene sequences. Subsequent repair of the gap would result in the restoration of the host allele. In the final case, flanking of the gene drive and associated transgenes by a direct repeat corresponding to the wild-type host allele renders all transgene sequences susceptible to loss via a form of DNA break repair known as single-strand annealing (SSA, figure 1*c*). In this case, a site-specific nuclease can be directed to generate a targeted DNA break, not in the host gene, but in the transgenic construct itself. This second nuclease could be an independently coded gene from that involved in gene drive, or the DNA break could simply be generated from the inclusion of an independent synthetic guide RNA, different from that required for a CRISPR-based gene drive. In either scenario, homology between the two repeated sequences promotes SSA-based repair following the double-stranded break, resulting in the loss of all transgene sequences and restoration of the host allele (figure 1*c*). In each of the three independent cases, the effect of the SEM is to trigger *in cis* removal of all transgene sequences while simultaneously generating a transgene-free allele that is resistant to future cleavage by the same CRISPR/Cas9-based gene drive. While the use of a recombinase would leave behind a scar that might perturb the activity of the host gene, silent nucleotide changes incorporated into either the transposon- or SSA-based approaches could preserve the wild-type amino acid sequence at the target gene and still provide resistance to further cleavage by the CRISPR/Cas9 gene drive.

**Figure 2.**
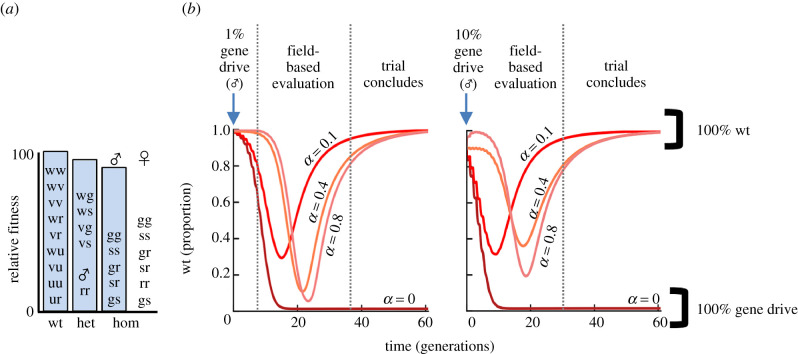
SEMs reverse potent gene drive systems. (*a*) Fitness penalties applied in the simulation for each genotype for a homing-based gene drive system targeting a gene critical for female fertility. (*b*) Proportion of transgene-free alleles after a single simulated release of gene drive containing individuals at 1% or 10% of a wild-type population when the selection for gene drive-resistant allele is not possible. Model outcomes for four SEM rates (*α* = 0, 0.1, 0.4, 0.8) are shown, all include an SEM failure rate of 1%.

To evaluate how such an SEM might affect the spread and persistence of a gene drive transgene in a randomly mating population, we modified previously developed deterministic models for homing-based gene drive [33] to incorporate a probability for both successful and failed transgene elimination. In total, the model considered six allele types (electronic supplementary material, figure S1A) and six rates of SEM (electronic supplementary material, figure S1B) that govern how each of the alleles can be generated or lost. Previous models [33,34] and accumulating biological data agree that when the fitness cost associated with disruption of a host gene by a gene drive transgene is small (electronic supplementary material, figure S2), resistance alleles arise and displace the invading gene drive. For a gene drive targeted to a non-essential gene, the addition of an SEM acting at just 10% efficiency is predicted to dramatically accelerate the displacement of gene drive alleles (electronic supplementary material, figure S2), which could be slowed, but not prevented, by increasing the SEM failure rate (electronic supplementary material, figure S3). While gene drive transgenes targeting non-essential genes are unlikely to move to field trials and thus are not likely to require additional containment mechanisms, these results encouraged us to pursue more difficult cases where such controls are needed. We note that as the probability of generating low-cost resistance alleles decreases, the expected persistence of a gene drive transgene in a population is expected to increase (electronic supplementary material, figure S4). However, the incorporation of an SEM prevented the fixation of such a strong gene drive transgene and rapidly restored wild-type genotypes across a wide range of efficiencies (10–80%, figure 2). *Dsx*-like gene drive transgenes were removed from the population even at an SEM breakdown rate of 10%; this was sufficient to avert complete population collapse (electronic supplementary material, figure S5). Importantly, the inclusion of an active SEM did not prevent the initial invasion of the target population by the gene drive transgene, but rapidly reversed its prevalence (temporal control).

**Figure 3.**
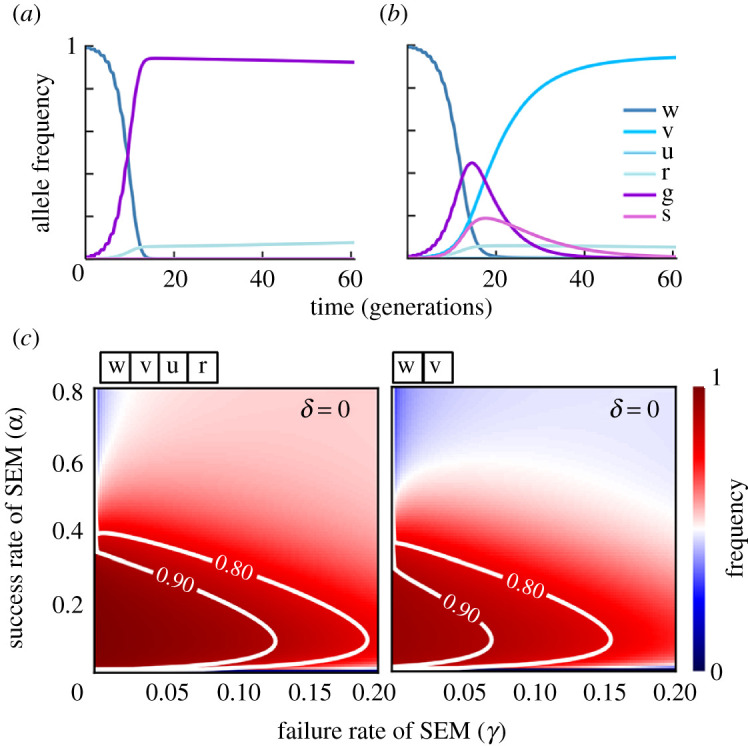
Self-elimination strategies are predicted to provide temporal control of gene drive transgenes over a broad parameter space, even when natural resistance alleles cannot be selected. Proportion of each allele in a simulated population after a single release of gene drive (genotype gg) males corresponding to 1% of the starting population when no-cost CRISPR-resistance alleles (u) cannot form (*δ* = 0) in the absence (*a*) or presence of a SEM (*b*). (*c*) Proportion of transgene-free alleles (w, wild-type; v, SEM-generated resistant; u, no-cost resistant; r, high-cost resistant) after 60 generations under a range of SEM (*α*) and SEM failure (*γ*) rates in the absence of natural resistance alleles (*δ* = 0).

To better understand the underlying dynamics, we calculated individual allele frequencies in the absence (figure 3*a*) or presence (figure 3*b*) of an SEM when naturally occurring resistance alleles cannot be selected for owing to their high fitness costs. Without the SEM, gene drive alleles rapidly dominate the population, with a small percentage of high-cost resistance alleles making up a consistent low-level minority. By contrast, no-cost resistance alleles (v) generated by the SEM quickly overtook gene drive alleles, which were lost from the population (figure 3*b*; electronic supplementary material, figure S5C). This was true for a broad range of rates for both SEM (0–80%) and SEM failure (0–20%), despite the absence of selection for natural resistance alleles (*δ* = 0), as the inclusion of an SEM led to the restoration of the population to a transgene-free status (figure 3*c*; electronic supplementary material, figure S5C). We conclude that incorporating an SEM approach into a homing-based gene drive transgene can potentially provide unprecedented control over the persistence of these invasive genetic elements while still allowing their temporary spread into a target population during field-based evaluation and risk assessment.

**Figure 4.**
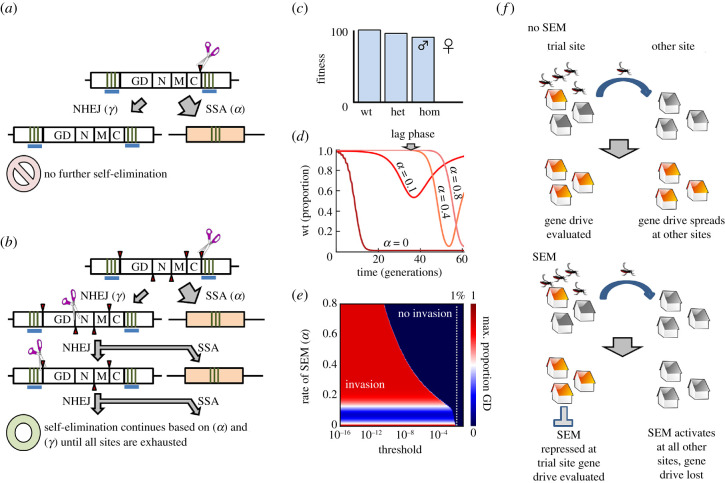
Self-elimination may provide spatial control of gene drive transgenes at low, but not arbitrarily low thresholds. (*a*) A single SEM failure through imperfect NHEJ-based repair at the nuclease recognition site. (*b*) The inclusion of multiple nuclease recognition sites (red arrows, *n* = 5) allows multiple independent attempts at self-elimination. Fitness parameters (*c*) used in simulated release (*d*) of gene drive-containing males at 1% of the population with five failures of the SEM required to create an SEM-resistant allele (s); the formation of no-cost resistant alleles (u) was not allowed (*δ* = 0). Model outcomes for four SEM rates (*α* = 0, 0.1, 0.4, 0.8) are shown, all include an SEM failure rate of 1%. Arrow indicates a lag phase where gene drive frequencies approach, but can never reach, zero. (*e*) If the proportion of gene drive alleles fell below the indicated threshold, it was considered lost, and the maximum proportion of transgenic individuals (from *T*_0_ to *T*_lost_ or, if never reached, *T*_0_ to *T*_end_) was calculated. (*f*) Potential spatial control provided by a SEM that was repressed conditionally during a contained field trial.

We next considered the potential for multiplexing to increase self-elimination efficiency and prevent gene drive invasion into sites outside of any potential trial area (spatial control), as currently proposed methods for spatial control of gene drive require multiple independently segregating transgenes, bioremediation or both [25–27]. In particular, SEMs based on a nuclease-induced double-stranded DNA break and SSA repair (figure 4*a*) could be multiplexed by simply increasing the number of nuclease recognition sites in the gene drive transgene (figure 4*b*). We again modelled a gene drive scenario based on the disruption of a gene critical for female fertility such as *dsx* (figure 4*c*), and this time allowed five independent attempts at transgene elimination. Multiplexing of the SEM substantially delayed, but never prevented, invasion of the gene drive transgene in the simulated population (figure 4*d*). As our model only allows allele frequencies to approach, but never actually reach zero, we considered that during the extended lag phase observed for even moderate values of SEM (0.4), the allele frequency of the gene drive transgene might fall so close to zero as to be considered practically zero. We plotted the maximum frequency of the gene drive transgene at any point during the simulation for arbitrary thresholds (not to be confused with the threshold for invasion of the gene drive transgene itself) down to 10^−16^, below each of which it was considered lost owing to a stochastic event (figure 4*e*). While a relatively crude method of introducing stochasticity, the inclusion of a multiplexed SEM reduced the frequency of the *dsx* gene drive transgene in the target population by up to 6–7 orders of magnitude below the initial release frequency. Altogether, these data suggest that at high rates (greater than 0.8), a multiplexed SEM may serve as a form of biocontainment for low-threshold gene drives (spatial control, figure 4*f*), while at lower rates (greater than 0–0.2), even a single SEM renders the gene drive essentially biodegradable (temporal control).

## 3. Discussion

The ability to develop powerful gene drive approaches not subject to genetic resistance selection reinforces the idea that it is essential to develop methods that provide precise control over them if these tools are to be successfully tested in field-based applications. Splitting a homing-based gene drive into two [9,26,35] or more [25] independently segregating gene cassettes is predicted to provide a layer of spatial control over the process of gene drive, as all fragments are required to catalyse transgene invasion. While advantageous, the use of multiple gene fragments to a single effect may complicate any risk assessment process, as any environmental impacts may need to be measured for all fragments not only on their own, but also in all possible combinations with every other fragment. Additionally, in such circumstances. while the act of gene drive is predicted to be temporally restricted, the transgene components themselves are not. Thus, to remove these transgenic sequences from a field population would require sustained inundative releases of wild-type individuals at the conclusion of any trial [27]. While not explicitly tested here, it may be possible to accelerate the removal of split drive components using the same SEM mechanisms as described here. The use of synthetic-resistance alleles released simultaneously with gene drive individuals has also been proposed as potential mitigation strategy [36]. However, such alleles could not control any gene drive transgenes that spread outside the trial site, and thus would also require a wave of remediation in the wake of an invading gene drive; logistically, this may not even be possible as gene drive-containing organisms can cross borders. The incorporation of an SEM allows the development of a single-component gene drive system (potentially simplifying risk assessment) while also providing a strong temporal limitation on the presence of the gene drive transgene in nature, without the need for remediative releases of non-transgenic individuals. While the use of a repressible lethal gene in combination with gene drive is conceptually similar [37], the strong selection pressure to lose such detrimental genes implies that when the safeguard fails, the gene drive could spread uninhibited. By contrast, the use of an SEM does not necessarily impose a strong fitness cost on the host, and the successful elimination of at least some gene drive transgenes creates a pool of resistance alleles in the target population that could prevent the spread of the gene drive as discussed below. We note that recombination systems such as Cre are highly efficient at catalysing the excision of transgenes in disease vector mosquitoes [38,39], *piggyBac* transposase can be excised and remobilized in malaria vectors [40], and SSA-based repair can result in transgene elimination in dengue vectors [41]. While the development and optimization of potential SEMs will no doubt require substantial effort, there is already substantial evidence that the technical aspects are tractable using existing technologies.

**Figure 5.**
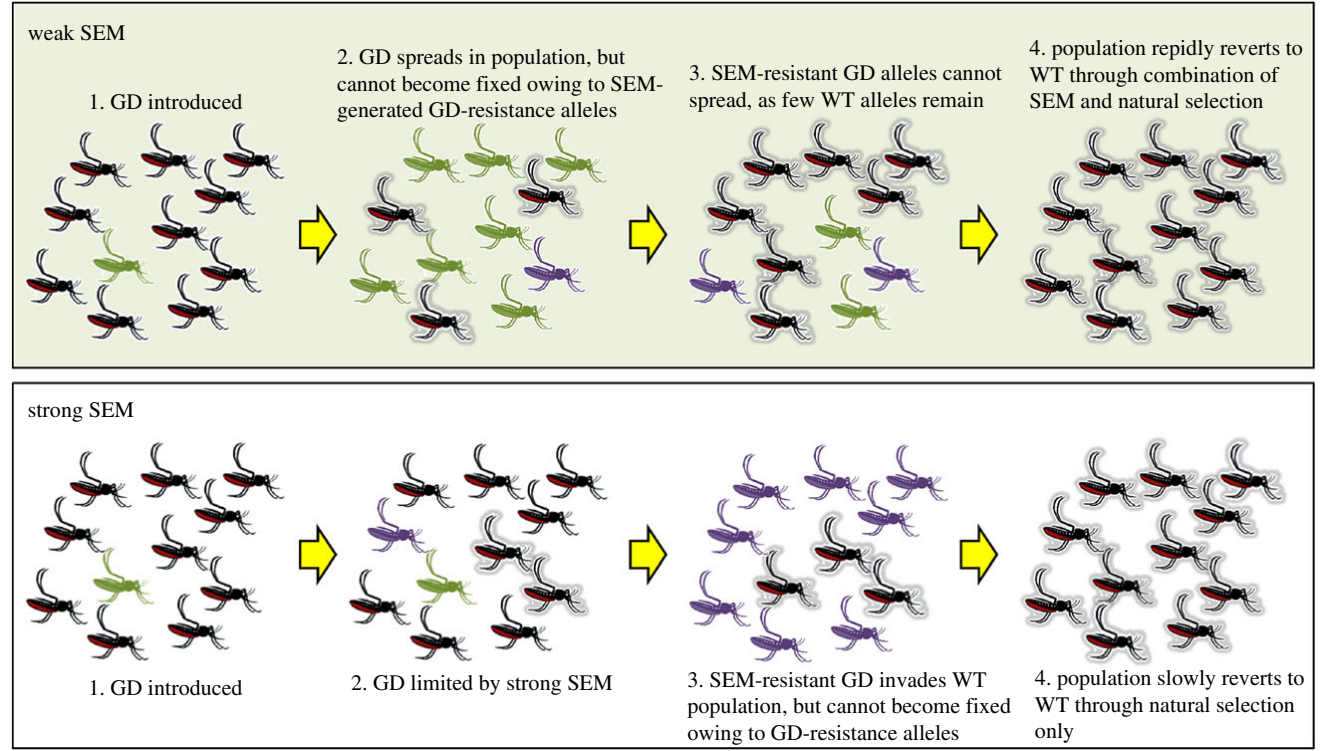
Rationale for why a weak SEM may outperform a strong SEM. GD, gene drive allele (g); GD-resistance allele (v); SEM-resistant GD allele (s). Colour of icons indicates genotype: WT (w: black, plain), GD (g: green), GD-resistant (v: black, glow), SEM-resistant GD (s: purple).

Just as CRISPR/Cas9 or other homing-based gene drive approaches that rely on homology-based repair can be thwarted by unwanted end-joining repair [14,16], SEMs that rely on DNA repair will eventually break down through the accumulation of end-joining based resistance alleles at the target site. Deleterious mutations in the nuclease or transposase can also be expected to arise, given sufficient time and a large enough population. Importantly, our modelling suggests that approaches based upon self-eliminating transgenes will be robust against permanent SEM failure at rates of up to 10%, much higher than the rate of spontaneous mutation in eukaryotic genomes. Likewise, whereas rates of homing may need to be greater than 75% for efficient gene drive [42], our models suggest that very low rates of transgene self-elimination (less than 10%) are more efficient than higher rates at controlling gene drive transgenes. While somewhat counterintuitive, we found that when the initial spread of the gene drive is efficient, the transgene effectively immunizes the target population against versions that have lost the SEM (figure 5). By contrast, when elimination of the transgene happens too rapidly, the population remains susceptible to SEM-resistant versions. Importantly, the effectiveness of the SEM is predicted to increase with the potency of the gene drive mechanism. That is, gene drive approaches targeting a critical gene where resistance allele selection is difficult were found to be easier to control using an SEM than gene drive approaches targeting non-essential genes. This is fortuitous, as it is the former that are in the greatest need of control, as the latter could potentially be controlled through the generation of natural resistance alleles.

The inclusion of an SEM and any associated repressible system for controlling it will increase the size of the cargo that would need to be copied during homing-based gene drive. Where the SEM is based on additional guide RNAs, these added sequences are likely to be negligible in relation to the size of the entire construct. Likewise, effective systems for repressing the SEM during field-based evaluation will need to be as compact as possible. SEM-based approaches for controlling homing-based gene drives also require the pre-programing of at least one no-cost resistance allele to prevent re-invasion of the gene drive transgene. Ideally, such a resistance allele would be sufficiently complex as to be unable to arise spontaneously (i.e. resistance to multiplexed guide RNAs), and with the added benefit of providing a diagnostic signal that self-elimination of the transgene had occurred. Given these limitations, it may be possible to adapt SEM approaches to other recently developed gene drive approaches [43,44]. The silent changes underlying the resistant allele could themselves become the target of a future gene drive approach, allowing reuse of the target gene while also providing strict spatial restrictions, as only those that had eliminated the first gene drive would be susceptible to the second. Importantly, the phenotypic characteristics of pre-determined resistance alleles can be evaluated empirically in laboratory and field-based settings independent of the gene drive transgene. Such experiments could determine any fitness costs associated with the resistance allele generated by the SEM, as the fitness of this allele must be higher than those possessing all possible gene drive alleles (with or without the SEM), so selection can act on the former at the expense of the latter. Inclusion of an SEM adds a substantial layer of predictability to gene drive experiments: rather than striving to develop gene drive approaches that are indestructible (and yet likely to fail in unpredictable ways), gene drive approaches can be designed to fail, but in a pre-evaluated and consistent manner.

Decision-making concerning any gene drive organism will require assessments of effects on target and non-target populations of sufficient scale and scope as to reveal potential hazards to surrounding ecosystems or human health. How to conduct such trials while preventing the long-term establishment of complete or partial gene drive transgenes remains unknown [13]. Given that any such field trial might end for political, social or financial reasons in addition to scientific ones, remedial releases of additional organisms as required by other approaches [9,27,45] may not be possible. Indeed, the requirement for remediation at the conclusion of any gene drive field trial poses clear challenges for the principles of fairness and justice in regard to the affected communities [13]. Put simply, it is not clear how a gene drive field trial can ever be fair to the communities involved if remediation is required, as these communities are not free to end their association with the research team at any time. That is, once the trial begins, only the research team can provide the infrastructure and personnel needed for remediation; otherwise, the cost and burden fall to the community itself. Thus, the inclusion of an SEM to set a strict time limit on the presence of a gene drive (or other) transgene in nature may very well be a prerequisite to the fair testing of gene drive technology and its potential acceptance by a sceptical public.

## 4. Material and methods

### (a) Model structure

For each of the gene drive mechanisms, we developed a system of delayed differential equations that predicted the number of offspring generated during each time step. Malthusian population growth was assumed with a daily time step through the models. Differential equations were concatenated and analysed using MATLAB 2017b. A single core with 8 GB of memory was sufficient for running MATLAB models to capture the proportions of wild-type individuals and allele progressions for all models. Parameter spaces for the remaining models used 112 cores with 392 GB of memory for up to 24 h from the Texas A&M University High Performance Research Computing (HPRC) Terra cluster for the computation of these parameter spaces. Model outputs were saved to a comma-separated values (.csv) file and plotted using Python 3.7.

The system dynamics models returned the number of adult and juvenile individuals of each genotype for every time step throughout the simulation. Initial model parameters are provided in the electronic supplementary material, table S1.

Using the fitness costs (*c*) associated with each genotype and sex (*i*), adult and juvenile mortality rates ($\mu_{A}$ and $\mu_{J}$, respectively) were adjusted such that the mortality rate could not be more than 1, giving

$$\mu_{A_{i}\;}=\;\frac{\mu_{A}}{\left(1-c_{i}\right)}\;\text{for}\;(1-c_{i})\geq \;\mu_{A},\;\text{otherwise}\;\mu_{Ai}=1,$$

$$\mu_{J_{i}\;}=\;\frac{\mu_{J}}{\left(1-c_{i}\right)}\;\text{for}\;(1-c_{i})\geq \;\mu_{J},\;\;\;\text{otherwise}\;\mu_{Ji}=1.$$

Mortality rates were applied at each time step, where the surviving number of adult individuals of each genotype *A_i_(T)* was calculated by reducing the number of adult individuals of each genotype at the previous time step *A_i_*(*T −* 1) by the mortality rate, such that

$$A_{i}(T)=(1-\mu_{A_{i}})A_{i}(T-1).$$

Juvenile mortality was applied at the time the juveniles became adults, where the number of juvenile individuals surviving the development period (*η*) was defined as

$$\;J_{i}(T-\eta ){\left(1-\mu_{J_{i}}\right)}^{\eta }.$$

Combining the surviving adults with the fully developed juveniles (also now adults), the number of adults with a particular genotype at time *T* can be defined as the number of adults surviving a single time increment (from time *T −* 1) and the number of surviving juveniles (from time T−η), such that

$$A_{i}(T)=(1-\mu_{A_{i}})A_{i}(T-1)+J_{i}(T-\eta ){\left(1-\mu_{J_{i}}\right)}^{\eta }.$$

The number of females with a particular genotype *F_i_* was directly used in calculating the number of offspring produced. Because males do not directly produce offspring, the proportion of adult males with a particular genotype *M_i_* was calculated such that

$$M_{i}=A_{M_{i}}\overset{n}{\underset{i=1}{\sum }}\frac{1}{A_{M_{i}}}.$$

Using the equations generated for the calculation of the number of offspring of each genotype, the fitness costs, initial input, self-elimination (*α*, *β*, *γ*), the probability of double-stranded break induction (*q*, 0.95) and the probability of homology-dependant repair (*p*, 0.95), the number of offspring created for each time step were calculated.

### (b) Equation generation

A two-dimensional matrix was generated of all the possible genotypes of females$\;(F_{i})$ and males$\;(M_{i})$. A third dimension was added to capture every possible outcome of offspring $\left(g_{i}\right)$. The value of each index within this three-dimensional matrix corresponded to the probability that the combination of the two parental genotypes would produce the respective offspring of the genotype. Iterating through all possible combinations of $\;F_{i}$, $\;M_{i}$ and $g_{i}$, a matrix of probabilities was generated. Once the matrix was fully populated, a string was concatenated with the parental genotypes and probability of producing an offspring, resulting in the form

$$F_{i}\;*\;\Psi (g_{i}\;|\;F_{i},M_{i})\;*\;M_{i}.$$

This was used in the calculation of the number of offspring in the system dynamics model. All combinations of parental genotypes to create a particular offspring genotype *k* were concatenated in the form

$$g_{i}=\;\overset{l}{\underset{\;j=1}{\sum }}\overset{n}{\underset{k=1}{\sum }}F_{j}\;*\;\Psi (g_{i}\;|\;F_{j},M_{k})\;*\;M_{k}.$$

Equations were simplified using MATLAB's *str2sym* function to reduce the additional computations necessary when referencing and calculating equations from the system dynamics model. To calculate the daily number of offspring of genotype *i* that were being produced, daily reproduction rates, sex ratio and fitness costs were additionally concatenated into the equation following the simplification of the equations, for females giving

$$\frac{\partial g_{i}}{\partial t}=\lambda \;*\;\sigma \;*\;(1-c_{i})\overset{l}{\underset{\;j=1}{\sum }}\overset{n}{\underset{k=1}{\sum }}[F_{j}\;*\;\Psi (g_{i}\;|\;F_{j},M_{k})\;*\;M_{k}]$$

and for males

$$\frac{\partial g_{i}}{\partial t}=\lambda \;*\;(1-\sigma )\;*\;(1-c_{i})\overset{l}{\underset{\;j=1}{\sum }}\overset{n}{\underset{k=1}{\sum }}[F_{j}\;*\;\Psi (g_{i}\;|\;F_{j},M_{k})\;*\;M_{k}].$$

## Supplementary Material

Supplemental Appendix

## Data accessibility

All data are available in the main text, electronic supplementary material or at: https://github.com/jozinzapletal/Making-gene-drives-biodegradeable.
