# Supplementary material for: Making gene drive biodegradable
Source: Philos Trans R Soc Lond B Biol Sci. 2020 Dec 28;376(1818):20190804. doi: 10.1098/rstb.2019.0804 (PMC7776940; doi:10.1098/rstb.2019.0804)
Supplement: Supplemental Appendix [file rstb20190804supp1.docx]

Supplementary Information for

Making gene drive biodegradable

Josef Zapletal, Neda Najmitabrizi, Madhav Erraguntla, Mark A. Lawley, Kevin M. Myles and Zach N. Adelman.

Correspondence to: [zachadel@tamu.edu](mailto:zachadel@tamu.edu)

**This PDF file includes:**

Supplementary Text

Figs. S1 to S5

Tables S1

Supplementary Text

Probability Calculations

Each of the gene-drive mechanisms differed in how the probabilities were determined. Since there was no HDR in the MEDEA and under-dominance mechanisms, the time at which the self-elimination occurred was equivalent whether it occurred in the parent or once the zygote was formed (the combination of the two parental alleles). In the CRISPR gene drive system, due to the multiple outcomes of a heterozygous *wg* or *ws* individual, the self-elimination mechanism was applied in the parent and the resulting gametes were then used in the formation of the offspring.

CRISPR

For each of the CRISPR scenarios, the self-elimination, DSB by Cas9, HDR, and NHEJ were assumed in the formation of gametes within the adults. As a result, the alleles that could be inherited by offspring from the parents was determined by the probabilities that each parent could contribute a particular gamete. For all scenarios, we assumed self-elimination would occur prior to the occurrence of a DSB.

CRISPR Single Self-Elimination Target Site with Single gRNA Target Site

In the case of a single gRNA target site with a single chance for self-elimination, six alleles (*w, v, g, s, u,* and *r*) were utilized creating 21 possible genotypes. The presence of a *g* allele allowed for self-elimination, producing *v, g,* or *s* alleles with probabilities α, ß, and γ, respectively. If a transgenic allele (*g* or *s*) was present with a *w* allele, a DSB could occur with probability *q*. The *v, u,* and *r* alleles have lost the gRNA target site and therefore cannot be cut by Cas9. The probabilities of inheritance are initially presented by Noble et al. [23] and modified here to include the self-eliminating mechanism.

The probability that gametes are passed from parents from offspring is defined as follows:

- Individuals with resistant alleles (*v, u, r*) and the wild-type allele *w* will produce resistant and wild-type gametes equiprobably such that we have

$$P_{wv,w}=P_{wu,w}=P_{wr,w}=P_{wv,v}=P_{wu,u}=P_{wr,r}=\frac{1}{2}$$

- Individuals with resistant alleles (*v, u, r*) and the permanently fixed transgenic allele *s* will produce resistant and permanently fixed transgenic gametes equiprobably such that we have

$$P_{vs,v}=P_{us,u}=P_{rs,r}=P_{vs,s}=P_{us,s}=P_{rs,s}=\frac{1}{2}$$

- Individuals with the resistant allele *v* with transgenic allele *g* will produce resistant gamete *v* through inheritance and self-elimination such that

$$P_{vg,v}=\frac{1+\alpha}{2}$$

- Individuals with wild-type allele *w* or resistant alleles *u* or *r* with transgenic allele *g* will produce resistant gamete *v* such that

$$P_{wg,v}=P_{ug,v}=P_{rg,v}=\frac{\alpha}{2}$$

- Individuals with resistant alleles *u* or *r* with transgenic allele *g* will produce resistant gametes *u* or *r* such that

$$P_{ug,u}=P_{rg,r}=\frac{1}{2}$$

- Individuals with resistant alleles (*v, u, r*) with transgenic allele *g* will produce transgenic gamete *g* when self-elimination and permanent fixation does not occur such that

$$P_{vg,g}=P_{ug,g}=P_{rg,g}=\frac{ß}{2}$$

- Individuals with resistant alleles (*v, u, r*) with transgenic allele *g* will produce permanently fixed transgenic gamete *s* when permanent fixation occurs such that

$$P_{vg,s}=P_{ug,s}=P_{rg,s}=\frac{\gamma}{2}$$

- Individuals with wild-type allele *w* and transgenic allele *g* will produce wild-type gamete *w* when successful self-elimination excision occurs or when self-elimination does not occur and no cutting of the target site occurs such that

$$P_{wg,w}=\frac{\alpha+ (1-q)(ß+\gamma)}{2}$$

- Individuals with wild-type allele *w* and transgenic allele *g* will produce transgenic gamete *g* when successful self-elimination excision does not occur or permanent transgene fixation does not occur. Through cutting and HDR, the transgenic material is copied onto the *w* allele such that

$$P_{wg,g}=\frac{(1+qp)(ß)}{2}$$

- Individuals with wild-type allele *w* and transgenic gamete *g* will produce resistant allele *u* when successful self-elimination excision does not occur or permanent transgene fixation occurs. Through cutting and NHEJ a functional resistant allele is produced such that

$$P_{wg,u}=\frac{(ß+\gamma)q(1-P)\delta}{2}$$

- Individuals with wild-type allele *w* and transgenic allele *g* will produce resistant gamete *r* when successful self-elimination excision does not occur or permanent transgene fixation occurs. Through cutting and NHEJ a nonfunctional resistant allele is produced such that

$$P_{wg,r}=\frac{(ß+\gamma)q(1-p)(1-\delta)}{2}$$

- Individuals with wild-type allele *w* and the permanently fixed transgenic allele *s* will produce wild-type gamete *w* if cutting does not occur such that we have

$$P_{ws,w}=\frac{1-q}{2}$$

- Individuals with wild-type allele *w* and the permanently fixed transgenic allele *s* will produce permanently fixed transgenic gamete *s* through cutting and HDR, when the transgenic material is copied onto the *w* allele such that we have

$$P_{ws,s}= \frac{1+qp}{2}$$

- Individuals with wild-type allele *w* and the permanently fixed transgenic allele *s* will produce resistant gamete *u* through cutting at the target site with probability *q* and NHEJ with probability *1-p*, repairing the cut to form a functional allele with probability *δ*, such that

$$P_{ws,u}=\frac{q\left( 1-p \right)\delta}{2}$$

- Individuals with wild-type allele *w* and the permanently fixed transgenic allele *s* will produce resistant gamete *u* through cutting and NHEJ, repairing the cut to form a function allele with probability δ, such that

$$P_{ws,r}= \frac{q\left( 1-p \right)(1-\delta)}{2}$$

Each of the alleles and probabilities for males and females were calculated and stored. By combining the two alleles (one from each parent) and multiplying the probabilities of the two alleles together, the probability that an offspring with a particular genotype could be created from two parents was obtained.

CRISPR Five Self-Elimination Target Sites with One gRNA Target Site

Using the structure of the single self-elimination cut with a single gRNA target site model, we introduced five self-elimination target sites modeled as *g_1­_-g_5_* (where *n =* 5)*,* allowing for five chances for a self-elimination cut to occur. Along with the wild-type and resistance alleles, this produces 55 possible genotypes. Transgene excision, no excision, and permanent transgene fixation follow a multinomial distribution, while Cas9 cutting, HDR, and NHEJ remain unchanged.

The probability that gametes are passed to offspring from parents are as follows:

- Individuals with resistant alleles (*v, u, r*) and the wild-type allele *w* will produce resistant and wild-type gametes equiprobably such that we have:

$$P_{wv,w}=P_{wu,w}= P_{wr,w}=P_{wv,v}=P_{wu,u}=P_{wr,r}=\frac{1}{2}$$

- Individuals with resistant alleles (*v, u, r*) and the permanently fixed transgenic allele *s* will produce resistant and permanently fixed transgenic gametes equiprobably such that we have:

$$P_{vs,v}=P_{us,u}=P_{rs,r}=P_{vs,s}=P_{us,s}=P_{rs,s}=\frac{1}{2}$$

- Individuals with wild-type allele *w* and the permanently fixed transgenic allele *s* will produce wild-type gamete *w* if cutting does not occur such that we have:

$$P_{ws,w}=\frac{1-q}{2}$$

- Individuals with wild-type allele *w* and the permanently fixed transgenic allele *s* will produce permanently fixed transgenic gamete *s* through cutting and HDR, when the transgenic material is copied onto the *w* allele such that we have:

$$P_{ws,s}=\frac{1+qp}{2}$$

- Individuals with wild-type allele *w* and the permanently fixed transgenic allele *s* will produce resistant gamete *u* through cutting at the target site with probability *q* and NHEJ with probability *1-p*, repairing the cut to form a functional allele with probability *δ*, such that:

$$P_{ws,u}=\frac{q\left( 1-p \right)\delta}{2}$$

- Individuals with wild-type allele *w* and the permanently fixed transgenic allele *s* will produce resistant gamete *u* through cutting and NHEJ, repairing the cut to form a function allele with probability δ, such that:

$$P_{ws,r}= \frac{q\left( 1-p \right)(1-\delta)}{2}$$

- Individuals with the resistant allele *v* with transgenic allele *g_i_* will produce resistant gamete *v* through inheritance and self-elimination such that:

$$P_{{vg}_{i}, v}=\frac{1+(1-\left( 1-\alpha\right)^{n-i})}{2}$$

- Individuals with the wild-type allele *w* or the resistant alleles *u* or *r* with transgenic allele *g_i_* will produce resistant gamete *v* through inheritance and self-elimination such that:

$$P_{{wg}_{i}, v}= P_{{ug}_{i}, v}=P_{{rg}_{i}, v}=\frac{(1-\left( 1-\alpha\right)^{n-i})}{2}$$

- Individuals with the resistant alleles *u* or *r* with transgenic allele *g_i_* will produce resistant gametes *u* or *r* such that:

$$P_{{ug}_{i}, u}=P_{{rg}_{i}, r}=\frac{1}{2}$$

- Individuals with the resistant alleles (*v, u, r*) with transgenic allele *g_i_* will produce transgenic gametes *g_i_* such that:

$$P_{{vg}_{i}, g_{i}}=P_{{ug}_{i}, g_{i}}=P_{{rg}_{i}, g_{i}}=\frac{ß^{n-i}}{2}$$

- Individuals with the resistant alleles (*v, u, r*) with transgenic allele *g_i_* will produce transgenic gametes *g_k_* such that:

$$P_{{vg}_{i}, g_{k}}=P_{{ug}_{i}, g_{i}}=P_{{rg}_{i}, g_{i}}=\frac{{\binom{n-i+1}{k-i}ß}^{n-k+1}\gamma^{k-i}}{2}$$

- Individuals with the resistant alleles (*v, u, r*) with transgenic allele *g_i_* will produce transgenic gamete *s* such that:

$$P_{{vg}_{i}, s}=P_{{ug}_{i}, s}=P_{{rg}_{i}, s}=\frac{\gamma^{n-i+i}}{2}$$

- Individuals with the wild-type alleles *w* with transgenic allele *g_i_* will produce wild-type gamete *w* such that:

$$P_{wg_{i},w}=\frac{\left( 1-\left( 1-\alpha\right)^{n-i} \right) + (1-q)(1-\alpha^{n-i})}{2}$$

- Individuals with the wild-type alleles *w* with transgenic allele *g_i_* will produce functional resistant gamete *u* such that:

$$P_{wg_{i},u}=\frac{{(1-\alpha)}^{n-i}q(1-p)\delta}{2}$$

- Individuals with the wild-type alleles *w* with transgenic allele *g_i_* will produce nonfunctional resistant gamete *r* such that:

$$P_{wg_{i},r}=\frac{\left( 1-\alpha\right)^{n-i}q(1-p)(1-\delta)}{2}$$

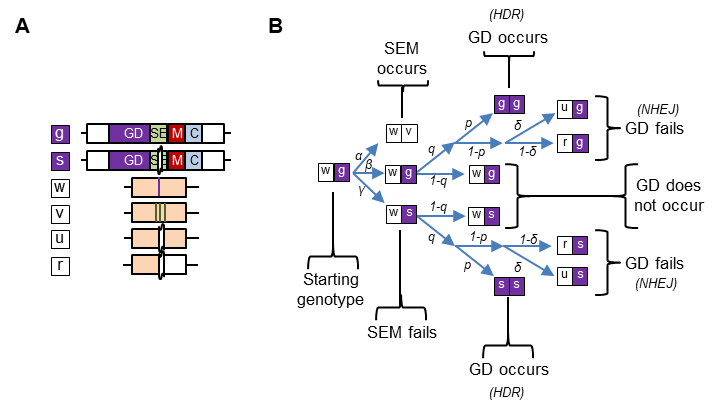


**Fig. S1. Modelling a self-eliminating gene drive.** (**A**) Six different allele types considered by our deterministic model. Two alleles contain the gene drive (GD), and either functional (g) or defective (s) self-elimination mechanism (SEM). Other alleles include wild-type, CRISPR-susceptible (w), wild-type, pre-determined CRISPR-resistant (v), CRISPR-resistant no cost (u), and CRISPR-resistant high cost (r). GD, gene drive; SE, self-elimination gene; M, marker gene; C, cargo gene (**B**) Structure and probabilities associated with our deterministic model and their relation to the six allele types : α, probability that self-elimination occurs; β, probability that the transgene is not altered by the SEM; γ, probability that the SEM mechanism breaks down without removing the transgene, with no chance for self-elimination to occur in any future generation; q, probability that the nuclease responsible for gene drive induces a double-stranded break at its target site on the homologous chromosome; p, the probability that the double-stranded break is repaired via homology-dependent repair (HDR).


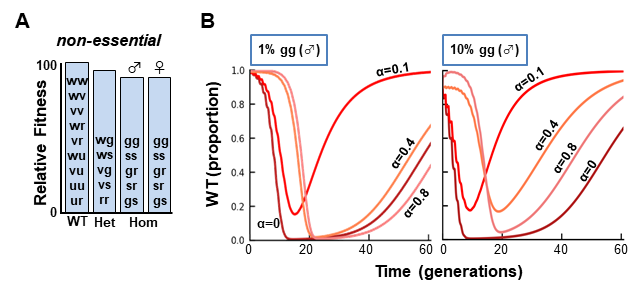


**Figure S2. Self-elimination mechanisms accelerate the reversal of gene drive systems without intervention.** (**A**) Fitness penalties applied to each potential genotype for a non-essential gene. (**B**) Proportion of transgene-free alleles after a single simulated release of gene drive containing individuals at 1% or 10% of a wild-type population when selection of no-cost resistance alleles is possible, at four SEM rates (α = 0, 0.1, 0.4, 0.8), with an SEM failure rate of 1%.


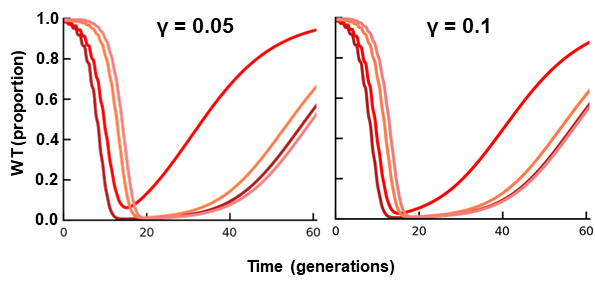


**Figure S3. Transgene self-elimination mechanisms are predicted to tolerate high failure rates.**  All plots are based on a starting population of gene drive containing individuals at 1% of the population and show four rates of transgene elimination (α=0,0.1, 0.4, 0.8) considering higher rates of failure of the self-elimination mechanism (0.05, 0.1).


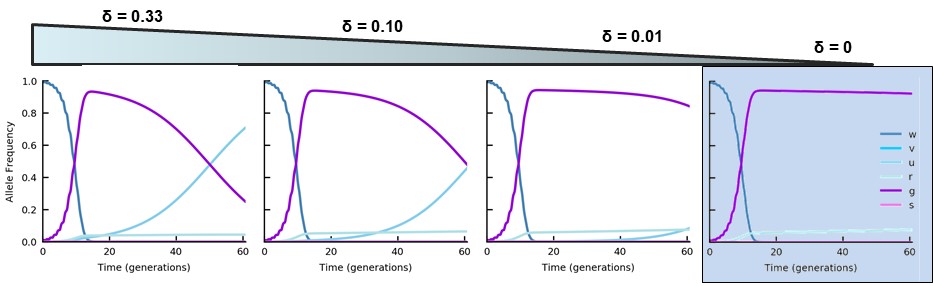


Fig. S4. As resistance allele formation becomes more difficult, gene drive transgenes last progressively longer in a simulated population. Proportion of each allele in a simulated population after a single release of gene drive (genotype gg) individuals corresponding to 1% of the starting population. Probability δ was set to 0.33, 0.1, 0.01 or 0 to simulate the increasing likelihood that a random indel results in a non-functional gene product. Shaded panel is from Figure 3A, but is included here for comparison purposes.


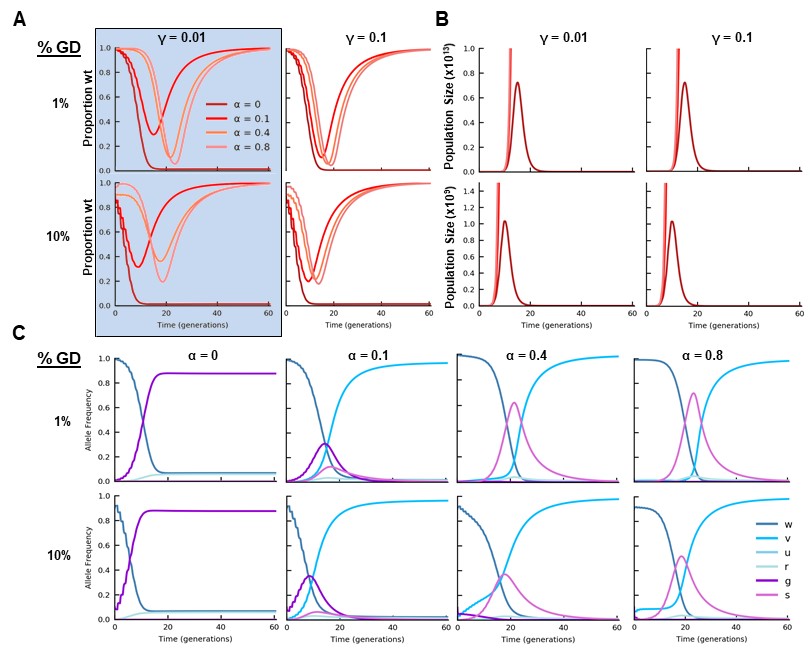


Fig. S5. Self-elimination strategies are predicted to remove a strong, sex-biasing gene drive and avert complete population elimination. Based on the approach described by Kyrou et al (2018), female genotypes rr, gr, sr, gg, ss, gs have fitness cost of 100%. Male genotypes gr, sr, gg, ss, gs have fitness cost of 10% and male genotype rr has a fitness cost 5%. We consider the gene drive to be active in both male and female germline with no chance of producing a functional resistance allele (δ = 0). (A) Proportion of transgene-free alleles (wt), absolute population size (B), and allele frequencies (C) after a single simulated release of gene drive (GD) individuals at 1% (top) or 10% (bottom) of a wild-type population at four different rates of transgene self-elimination (α = 0, 0.1, 0.4, 0.8). For (A + B), simulations using γ = 0.01 and γ = 0.1 are shown; For (C), γ = 0.01. Shaded panels are from Figure 2B and are printed here for comparison purposes.

Table S1. Variable Definitions

| **Variable** | **Description** | **Value** |
| --- | --- | --- |
| λ | Female reproduction rate (per day) | 7 |
| σ | Proportion of female offspring | 0.5 |
| $c_{i}$ | Fitness cost of genotype *i* | Varies |
| $\mu_{A}$ | Adult mortality rate (per day) | 0.3 |
| $\mu_{J}$ | Juvenile mortality rate (per day) | 0.03 |
| η | Development time (in days) | 12 |
